# Genome-wide association study of seed coat color in sesame (*Sesamum indicum* L.)

**DOI:** 10.1371/journal.pone.0251526

**Published:** 2021-05-21

**Authors:** Chengqi Cui, Yanyang Liu, Yan Liu, Xianghua Cui, Zhiyu Sun, Zhenwei Du, Ke Wu, Xiaolin Jiang, Hongxian Mei, Yongzhan Zheng

**Affiliations:** 1 Henan Sesame Research Center, Henan Academy of Agricultural Sciences, Zhengzhou, Henan, China; 2 Nanyang Academy of Agricultural Sciences, Nanyang, Henan, China; 3 Zhumadian Academy of Agricultural Sciences, Zhumadian, Henan, China; 4 College of Life Sciences, South China Normal University, Guangzhou, Guangdong, China; New South Wales Department of Primary Industries, AUSTRALIA

## Abstract

Sesame (*Sesamum indicum* L.) is an important and ancient oilseed crop. Sesame seed coat color is related to biochemical functions involved in protein and oil metabolism, and antioxidant content. Because of its complication, the genetic basis of sesame seed coat color remains poorly understood. To elucidate the factors affecting the genetic architecture of seed coat color, 366 sesame germplasm lines were evaluated for seed coat color in 12 environments. The genome-wide association studies (GWAS) for three seed coat color space values, best linear unbiased prediction (BLUP) values from a multi-environment trial analysis and principal component scores (PCs) of three seed coat color space values were conducted. GWAS for three seed coat color space values identified a total of 224 significant single nucleotide polymorphisms (SNPs, *P* < 2.34×10^−7^), with phenotypic variation explained (PVE) ranging from 1.01% to 22.10%, and 35 significant SNPs were detected in more than 6 environments. Based on BLUP values, 119 significant SNPs were identified, with PVE ranging from 8.83 to 31.98%. Comparing the results of the GWAS using phenotypic data from different environments and the BLUP values, all significant SNPs detected in more than 6 environments were also detected using the BLUP values. GWAS for PCs identified 197 significant SNPs, and 30 were detected in more than 6 environments. GWAS results for PCs were consistent with those for three color space values. Out of 224 significant SNPs, 22 were located in the confidence intervals of previous reported quantitative trait loci (QTLs). Finally, 92 candidate genes were identified in the vicinity of the 4 SNPs that were most significantly associated with sesame seed coat color. The results in this paper will provide new insights into the genetic basis of sesame seed coat color, and should be useful for molecular breeding in sesame.

## Introduction

Sesame (*Sesamum indicum* L., 2n = 2x = 26), which belongs to the *Sesamum* genus of the Pedaliaceae family, is one of the earliest domesticated crops [1]. It is mainly planted in tropical and subtropical regions in Asia, Africa, and South America [2]. Compared with the seeds of other main oil crops, e.g., rapeseed (*Brassica napus*), soybean (*Glycine max*), peanut (*Arachis hypogaea*) and olive (*Olea europaea*), sesame seeds not only have the highest oil content, but also are rich in proteins, vitamins, and specific antioxidants such as sesamin and sesamolin [3, 4]. Because of its high oil quality and high nutritive value, sesame seed is regarded as ‘the queen of oil seeds’ and one of the best choices for health foods [5].

Seed coat color is one of the most important agronomic traits of sesame. It is related to biochemical functions involved in protein and oil metabolism, antioxidant content, and disease resistance [6]. The natural color of mature sesame seeds is diverse, varying from black to white through different intermediates such as gray, dark brown, brown, pale brown, yellow and dirty white [1]. In general, pale-colored sesame seeds contain more oil than dark-colored ones [6, 7]. Therefore, white sesame seeds are usually used to produce oil, and black sesame seeds are favored as food and medication in China. Significant attention has been paid to the inheritance of seed coat color in sesame. Some early classical genetic studies have suggested that sesame seed coat color is determined by two genes [8, 9], while other reports have indicated that the genetic basis of sesame seed coat color is far more complex, which may involve multiple genes and their interactions [10, 11]. In recent years, the genotyping load and cost has been significantly reduced by the next-generation sequencing (NGS) technologies [12], several high-density genetic maps have been developed and a large number of quantitative trait loci (QTLs) for agronomically important traits have been identified in sesame [13–17], including QTLs for seed coat color [6, 15, 18]. However, QTL mapping efforts using the segregated progeny of a bi-parental cross only enable the detection of a subset of loci/alleles within the crop, and offer limited resolution owing to the small number of informative recombination events between linked genetic loci [19]. As an alternative approach to traditional QTL analysis, the genome-wide association study (GWAS), taking advantage of both the wide phenotypic variation and the high number of historical recombination events in natural populations, has been used for dissecting complex traits in crop species [20, 21], such as rice, maize, soybean, cotton, and rapeseed [22–26]. As an orphan or neglected crop, GWAS analysis in sesame is still limited. Wei et al. [27] re-sequenced 705 diverse sesame germplasm accessions and performed a comprehensive GWAS on 56 agronomic traits for the first time. Using a subset of 400 accessions from the above population, Dossa et al. [28] performed a large-scale GWAS on five traits related to drought tolerance.

In this study, seed coat color of an association-mapping panel comprising 366 sesame germplasm accessions was measured in 12 environments, and 42,781 SNPs were developed by using specific-locus amplified fragment sequencing (SLAF-seq). By performing a large-scale GWAS on seed coat color, significantly associated SNPs and candidate genes were explored. These SNPs and candidate genes will play important roles in understanding the genetic basis of seed coat color in sesame.

## Materials and methods

### Plant materials and experiment design

In a previous study, 366 diverse sesame lines were selected from the Henan Sesame Research Center (HSRC) sesame germplasm collection, and were assembled into an association-mapping panel [29]. In this study, the panel was used for seed coat color evaluation and marker-trait association analysis.

The association-mapping panel was grown at four locations in China for two to four years: Nanyang (NY, E112.52°, N33.00°), from 2013 to 2014; Pingyu (PY, E114.63°, N32.97°), from 2013 to 2016; Shangqiu (SQ, E115.65°, N34.45°), from 2013 to 2014; and Sanya (SY, E109.50°, N18.25°), from 2012 to 2015. Field experiments were arranged by a randomized complete block design, with two replications under each environment. Each accession was grown in a plot with 23–25 plants in a single row, with a distance of 0.15 m between plants within each row and 0.4 m between rows.

### Measurement of seed coat color and statistical analysis

Sesame seeds were harvested from five randomly chosen plants in each row at maturity, and were used to evaluate the seed coat color. Seed coat color was scored using a HunterLab colorimeter (ColorFlex EZ, Hunter Associates Laboratory Inc., Virginia, USA), and decomposed into L, a, and b color space values. The L-value represents brightness (black to white, 0 for black, 100 for white), the a-value represents the color from red to green (positive represents red, negative represents green), and the b-value represents the color from yellow to blue (positive represents yellow, negative represents blue) [30]. Descriptive statistics for sesame seed coat color value for each environment, were computed using the PROC UNIVARIATE procedure (*α* = 0.01) of SAS 8.02 software (SAS Institute, Cary, NC, USA). Best linear unbiased predictions (BLUPs) were used to estimate seed coat color values across multiple environments using the R [31] package “lme4” [32]. The BLUP model for the phenotypic trait was *y_ijk_* = *μ*+*G_i_*+*E_j_*+(*GE*)_*ij*_+*B*_*k*(*ij*)_+*ε_ijk_*, where *μ* is the total mean, *G_i_* is the genotypic effect of the *i*th genotype, *E_j_* is the effect of the *j*th environment, (*GE*)_*ij*_ is the interaction effect between the *i*th genotype and the *j*th environment, *B*_*k*(*ij*)_ is the effect of replication within the *j*th environment, and *ε_ijk_* is a random error following $N(0,\;\sigma_{e}^{2})$ [33]. The analysis of variance (ANOVA) was performed using QTL IciMapping V4.0 [34]. Broad sense heritability was calculated as: $H^{2}=\sigma_{G}^{2}/(\sigma_{G}^{2}+{(\sigma }_{GE}^{2}/k)+(\sigma_{\varepsilon }^{2}/rk))$, where $\sigma_{G}^{2}$ is the genotypic variance, $\sigma_{GE}^{2}$ is the genotype by environment variance, $\sigma_{\varepsilon }^{2}$ is the residual variance, *k* is the number of environments, and *r* is the number of replications [33]. Principal component analysis (PCA) can transform a set of correlated variables into a substantially smaller set of uncorrelated variables as principal components (PCs), which can capture most information from the original data [35]. Borcard et al. [36] recommended that the variables used in the PCA should be scaled to zero-mean and unit-variance. Therefore, PCA for three color space values was performed using R function "prcomp" with the setting "scale = TRUE" [31]. The first 2 PCs which explained 93%~97% of the total variance in different environments, were retained for GWAS.

### Marker-trait association analysis

In a previous study, the association-mapping panel was genotyped by using SLAF-seq, and 89,924 high quality SNPs (minor allele frequency (MAF) ≥ 0.01 and integrity ≥ 0.7) were identified [29]. In this study, to avoid the possible false SNP affecting the result of GWAS, a set of 42,781 SNP markers with a MAF ≥ 0.05 and integrity ≥ 0.7 was used to perform marker-trait association analysis. PCA matrix of the 42,781 SNPs was performed using the GCTA software [37]. The kinship (K) matrix was estimated using Tassel 5.0 software [38]. Marker-trait association analysis was performed for three color space values, BLUP values and two PCs of color space values using mixed linear models (PCA+K model) implemented in Tassel 5.0 software [38]. In the PCA+K model, the mixed linear model correcting for both PCA-matrix and K-matrix, were employed to reduce errors from population structure and relative kinship. The uniform Bonferroni threshold was used for the significance of associations between SNPs and traits at the significance level of 0.01. In this study, the threshold was −log_10_(0.01/42,781) ≈ 6.6 where 42,781 is the number of SNP markers. Manhattan and QQ plots were drawn using the R package “qqman” [39].

### Candidate gene prediction

To define the regions of interest for selection of potential candidate genes, the LD blocks, in which flanking SNP markers had strong LD (*r*^*2*^ > 0.6), were defined as the candidate gene regions [40]. All genes within the same LD block (*r*^2^ > 0.6) were considered as candidate genes. For significant SNPs outside of the LD blocks, the 99 kb (the LD decay distance) flanking regions on either side of the markers were used to identify candidate genes [29]. LD heatmaps surrounding peaks in the GWAS were constructed using the R package “LDheatmap” [41].

## Results

### Phenotypic variations of sesame seed coat color

**Fig 1:**
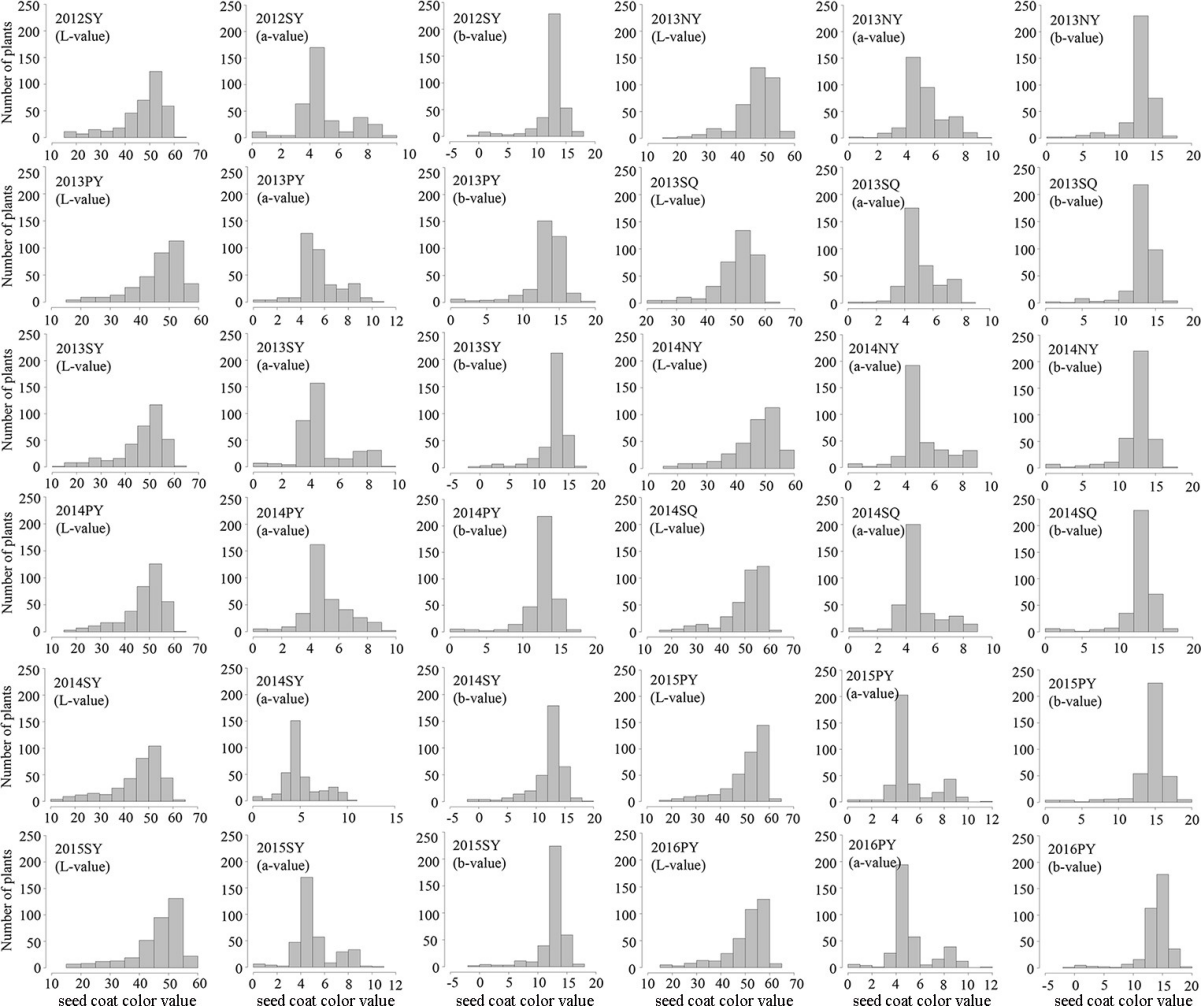
Histograms for the frequency distribution of three color space values (L-value, a-value and b-value).

**Fig 2:**
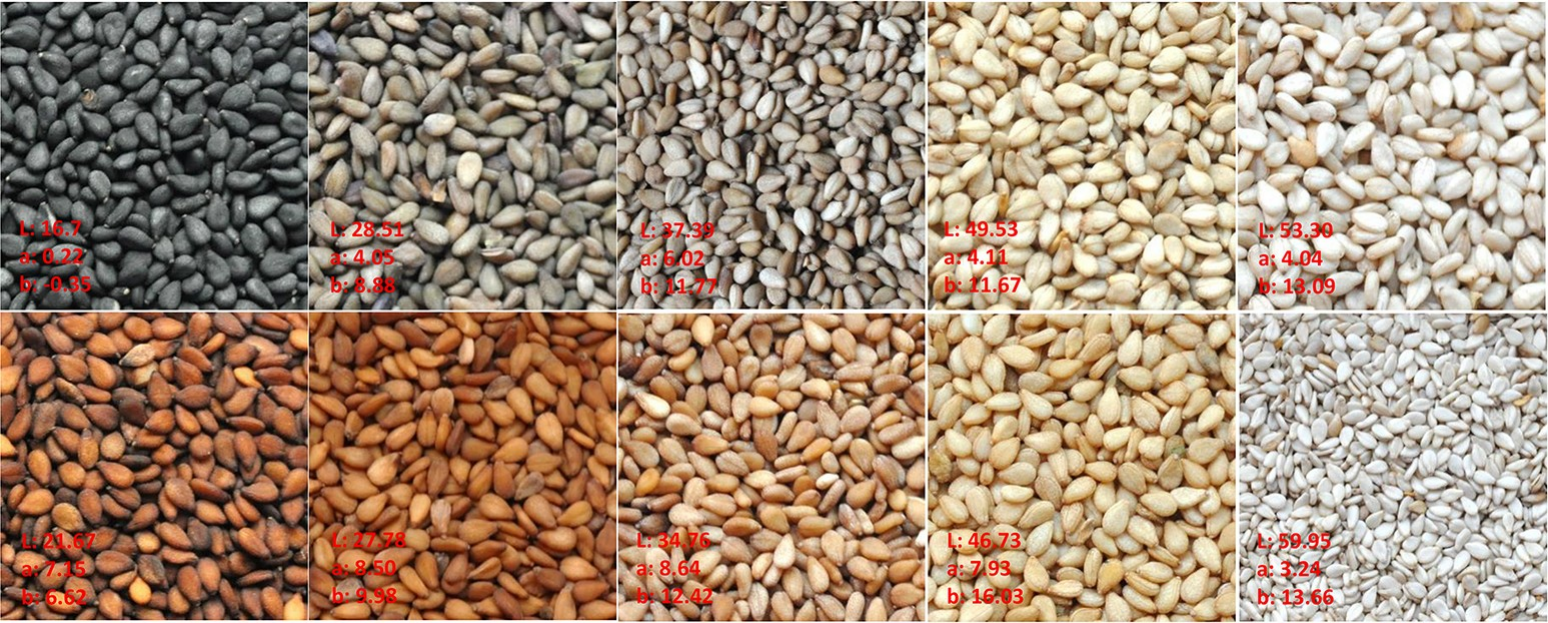
Seed coat color variation in sesame association panel.

To evaluate the phenotypic variation of seed coat color in the sesame association panel, three color space values (L-value, a-value, and b-value) in each environment and BLUP values across multiple environments were analyzed (Fig 1 and S1 Fig). Descriptive statistics for seed coat color were presented in S1 Table. The sesame association panel exhibited wide variations in seed coat color. The L-value exhibited a wide range of 10.53 to 63.40, with the coefficient of variation (CV) ranging from 14.08 to 22.94% among different environments. Similarly, the a-value ranged from 0.08 to 11.22, with CV ranging from 24.07 to 37.40%, and the b-value ranged from -0.47 to 18.75, with CV ranging from 15.51 to 24.50%. Because L-value represents brightness ranging from black to white (0 for black, 100 for white), a-value represents the color from red to green (positive represents red, negative represents green), and b-value represents the color from yellow to blue (positive represents yellow, negative represents blue), the measured values and distributions indicate that black, white, red, and yellow are predominant in the sesame seed coat color, which is consistent with the observation that the seed coat color distributions in the association panel (Figs 1 and 2 and S1 Fig). ANOVA was performed to reveal the effects of G (genotypes), E (environment) and G × E (interaction between G and E) for seed coat color trait in multi-environments. The results showed that there were highly significant differences among G, E, and G × E (P < 0.01). The broad-sense heritability of the L-value was calculated to be 98.16%, while the broad-sense heritability of the a-value and b-value was 97.55% and 96.88%, respectively.

PCA was performed for three space color values to investigate the relationships among three space color value variables. PC1 explained 56%~65% of the trait variances in different environments, and three space color values showed high negative loadings on PC1. This result suggested that seed coat color with high PC1 scores exhibited samll values for L-value, a-value and b-value. PC2 explained 34%~43% of the trait variances. Cumulative Proportion of variances for PC1 and PC2 were 93%~97%, and the PCA results were consistent with each other across different environments (S2 Table), suggesting that PC1 and PC2 can be used as quantitative indices to characterize sesame seed coat color.

### Genome-wide association analysis for sesame seed coat color

**Fig 3:**
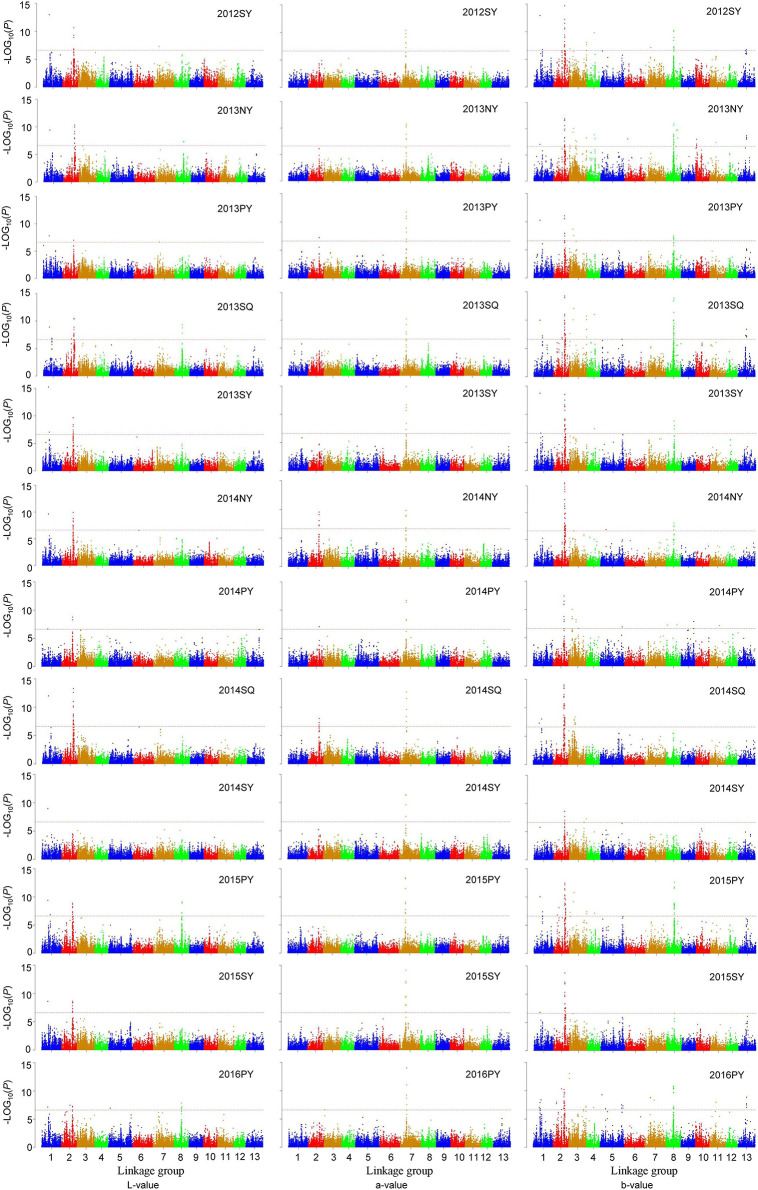
Genome-wide association studies (GWAS) of seed coat color in twelve environments. The red horizontal dashed lines indicate the genome-wide significance threshold (*P* < 2.34 × 10^−7^).

To uncover the genotypic variation of seed coat color in sesame, GWAS were performed for three color space values from different environments and BLUP values across all environments. Using three color space values, a total of 224 significant SNPs (*P* < 2.34×10^−7^) were identified in 12 environments (Fig 3), and the *R*^*2*^, the phenotypic variation explained (PVE) by SNPs, ranged from 1.01% to 22.10%. As shown in quantile-quantile plots (S2 Fig), the genomic inflation was considerably controlled. Among 224 significant SNPs, 35 were detected in more than 6 environments, 24 were detected in more than 8 environments, and 14 were detected in more than 10 environments (S3 Table). Using BLUP values, 119 significant SNPs were identified, with PVE ranging from 8.83 to 31.98% (S3 Fig). Comparing the results of the GWAS using phenotypic data from different environments and the BLUP values, all significant SNPs detected in more than 6 environments were also detected using the BLUP values (S3 Table).

Regarding L-value, 38 significant SNPs were detected on 5 linkage groups (LGs), with PVE ranging from 8.75% to 21.90%. Among these SNPs, 24 were detected using the BLUP values of L-value. The most significant SNP S1_6648896 on LG1 was detected in all 12 environments and was also detected using the BLUP values. On LG2, 8 multi-environment significant SNPs (S2_12167303, S2_12178804, S2_12178823, S2_12194998, S2_12232894, S2_12232938, S2_12447358, S2_12247409) were significantly associated with L-value in 7, 8, 8, 8, 7, 10, 8, and 9 environments and were also detected using the BLUP values (S3 Table). Regarding a-value, 17 significant SNPs were identified on LG2, LG3 and LG7, and 9 were detected using the BLUP values of a-value. Of all the significant SNPs, S7_6839839 was detected in all 12 environments and was also detected using the BLUP values, (S3 Table). Regarding the b-value, 169 significant SNPs distributing on LG1, LG2, LG3, LG4, LG5, LG6, LG7, LG8, LG9, LG10, LG11 and LG13 were identified, with PVE ranging from 8.68% to 31.35%. The Manhattan plots showed that 3 peaks on LG1, LG2, and LG8 were repeatedly detected in more than 6 environments and were also identified using BLUP values of b-value. Nine significant SNPs were detected on LG1. The SNP S1_6648896 with the lowest *P* value on LG1 was detected in 9 environments and was also detected using BLUP values. Seventy significant SNPs were detected on LG2. S2_12168663 and S2_12337057 were both detected in 7 environments. S2_12336812 was detected in 8 environments. S2_12167303 and S2_12247358 were detected in 9 environments. S2_12026452, S2_12178804, S2_12178823 and S2_12194998 were detected in 10 environments. S2_12015779, S2_12015820 and S2_12247409 were detected in 11 environments. S2_12232894 and S2_12232938 were detected in 12 environments. These 14 SNPs were also detected using BLUP values. On LG8, 4 multi-environment significant SNPs (S8_7910606, S8_8220220, S8_8311600, S8_8313501) were significantly associated with b-value in 7, 6, 6, and 7 environments and were also identified using BLUP values (S3 Table).

GWAS for PC1 and PC2 identified 197 significant SNPs (P < 3.3×10^−7^); however, significant SNPs were not detected for PC3 (S4 Fig; S4 Table), which indicated that PC3 might be composed of nongenetic factors. The quantile-quantile plots were shown in S5 Fig. Among 197 significant SNPs, 30 were detected in more than 6 environments, 19 were detected in more than 8 environments, and 14 were detected in more than 10 environments. For PC1, the GWAS results were consistent with those for L-value and b-value. One hundred and eighty-eight significant SNPs were identified on 12 LGs, explaining 8.68–33.93% of the phenotypic variation. Four peaks on LG1, LG2, LG4, and LG8 were repeatedly detected in more than 6 environments. The most significant SNP S1_6648896 on LG1 was repeatedly detected in 9 environments, explaining 12.93%~20.51% of the phenotypic variation. Nineteen significant SNPs on LG2 were indentified in more than 6 environments. The most significant SNP S2_12232938 on LG2 with PVE of 11.95~33.93% was detected in 12 environments. The most significant SNP S4_7766099 on LG4 was repeatedly detected in 6 environments, and explained 9.47%~15.26% of the phenotypic variation. Three significant SNPs on LG8 were detected in more than 6 environments. The most significant SNP S8_8313501 on LG8 was repeatedly detected in 8 environments, and explained 9.47%~15.26% of the phenotypic variation. The GWAS results for PC2 were consistent with those for a-value. Six significant SNPs on LG7 were detected in more than 6 environments. The most significant SNP S7_6839839 was repeatedly detected in 12 environments, and explained 14.14%~26.18% of the phenotypic variation.

### Candidate genes associated with sesame seed coat color

**Fig 4:**
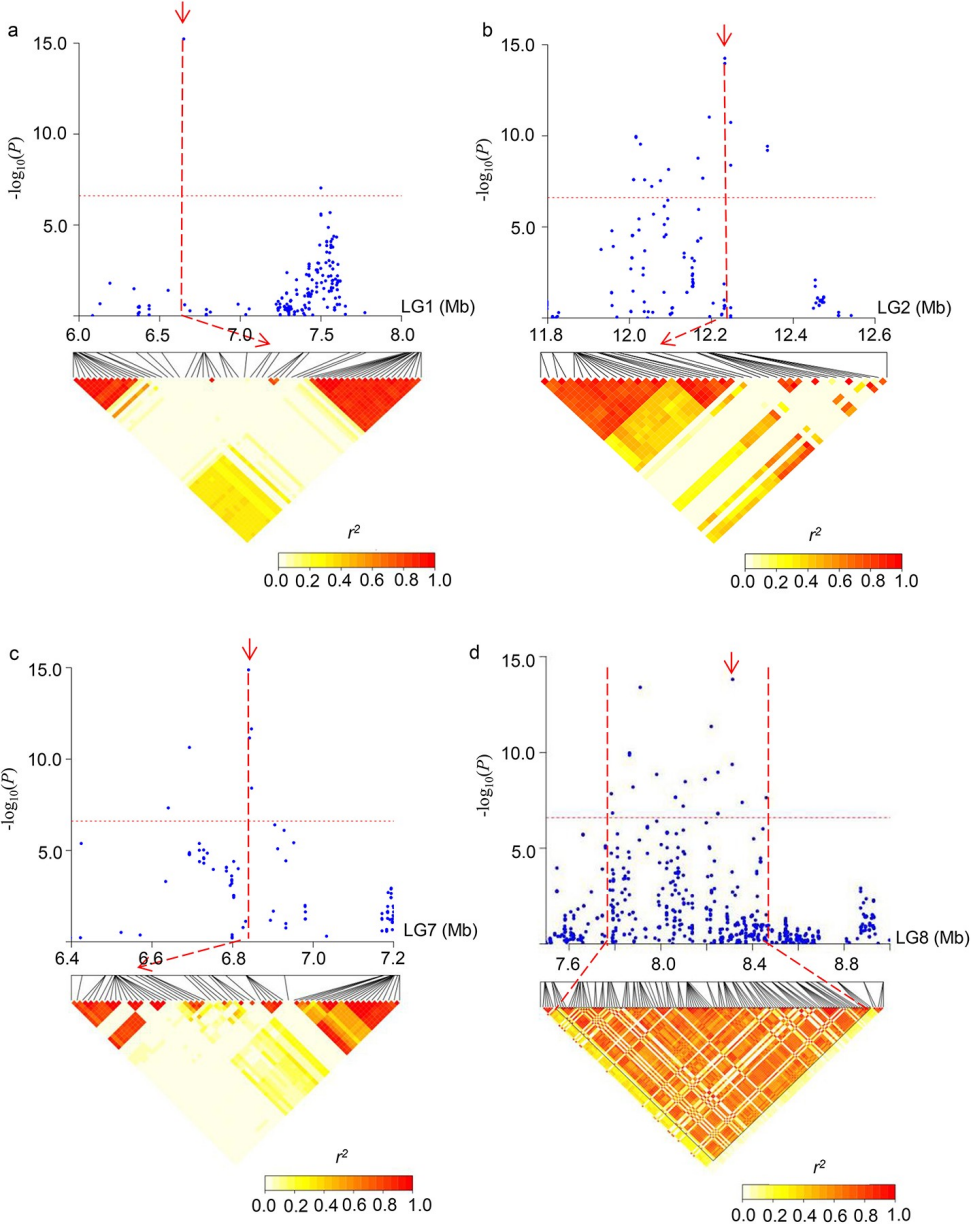
**Local Manhattan plot (top) and LD heatmap (bottom) surrounding each peak on different linkage groups.** (a) LD heatmap on LG1. The red arrow denotes the SNP S1_6648896; (b) LD heatmap on LG2. The red arrow denotes the SNP S2_12232938; (c) LD heatmap on LG7. The red arrow denotes the SNP S7_6839839; (d) LD heatmap on LG8. The red arrow denotes the SNP S8_8313501.

To predict the putative genes associated with sesame seed coat color, we focused on the most reliable and stable peaks on different LGs, including S1_6648896, S2_12232938, S7_6839839 and S8_8313501 (Fig 4). The haplotype analysis showed that the SNPs S1_6648896, S2_12232938 and S7_6839839 were all in genomic regions that were in state of linkage equilibrium, while S8_8313501 was involved in a 213-kbp LD block. Within the LD block (S8_8313501), or 99 kbp either side of the SNPs (S1_6648896, S2_12232938 and S7_6839839), a total of 21, 20, 31 and 20 genes were identified, respectively (S5 Table). Of the 92 genes, 26 had no definite annotation concerning their biological functions, and 12 were annotated as putative or probable proteins. The remaining 54 genes had domains of known functions. Gene ontology (GO) analysis indicated that 40, 39 and 31 genes were involved in the cellular component category, the molecular function category and the biological process category, respectively. In the cellular component category, these genes were grouped into cell (39 genes), cell part (39 genes) and organelle (36 genes) subcategories. Within the molecular function category, the majority of genes were involved in catalytic activity (14 genes), binding (15 genes), transcription regulator activity (6 genes). In the biological process category, most gene were annotated to metabolic process (23 genes), cellular process (31 genes), response to stimulus (20 genes).

## Discussion

GWAS has become an efficient and powerful tool at identifying genetic variations and loci responsible for the agronomically important traits. In 2015, a GWAS of oil quality and agronomic traits with 705 sesame lines identified several causative genes, demonstrating the feasibility of GWAS in sesame [27]. In the present study, the panel of sesame accessions with wide geographic distribution, plentiful phenotype variation, sufficient genetic variation and weak population structure is advantageous for GWAS implementation [29]. However, the reliability of GWAS is usually disturbed by phenotypic variance associated with the environment. Multi-environment analysis and unbiased predictions are practical ways to correct for this error [25]. The trait experiments were performed at four sites, which belong to three climate classifications, temperate monsoon climate (PY and SQ), subtropical monsoon climate (NY), and tropical marine monsoon climate (SY). Among four sites, there are large differences in geographic position and climate. ANOVA showed that significant variations were observed in G, E and G×E. This result suggested that sesame seed coat color was controlled by the genetic, environment effect and their interaction. Then, GWAS for coat color traits were performed in 12 environments, and many significant SNPs were only detected in a specific environment. However, the SNPs detected in more than 6 environments were detected using BLUP values in a multi-environment trial analysis. These multi-environment SNPs are reliable and will be used for further analysis. Therefore, the multi-environment trial analysis could effectively avoid influences from the environments, and is the way forward in the study of complex quantitative traits.

PCA is an effective approach for collecting information from complex, multiple traits that are highly correlated; furthermore, it is valuable for extracting underlying factors for traits by dimension reduction [35]. As PC scores represent integrated variables, they can result in robust, reliable GWAS results [35]. In this study, PCA on three space values (L-value, a-value and b-value) revealed that PC1 captured 56%~65% of variations for all values, PC2 captured 34%~43% of variations for L-value and a-value. Cumulative Proportion of variances for PC1 and PC2 were 93%~97% (S2 Table). Thus, PC1 and PC2 are good indicators for sesame seed coat color. Using the three color space values, 224 significant SNPs (*P* < 2.34×10^−7^) were identified. After combining the same SNPs associated with different seed coat color values (L-value, a-value and b-value), 185 SNPs were remained. Using the PC scores (PC1 and PC2) for GWAS, 201 significant SNPs associated with PCs were identified. The GWAS results for PC1 and PC2 were consistent with those for three color space values, indicating PC1 and PC2 can represent three space color values to perform GWAS.

To further confirm these significant SNPs associated with seed coat color in this paper, we compared our GWAS results with QTLs from previous linkage studies. Wang et al. [15] identified 4 QTLs (*qSCa-4*.*1*/*qSCb-4*.*1*/*qSCl-4*.*1*, *qSCa-8*.*1*/*qSCb-8*.*1*/*qSCl-8*.*1*, *qSCl-8*.*2*, and *qSCb-11*.*1*/*qSCl-11*.*1*) for seed coat color in a RIL population. Most of QTLs (3/4 QTLs) were verified by significant SNPs in the present study. Eighteen significant SNPs on LG2 were mapped to the confidence interval of the QTL *qSCa-4*.*1/qSCb-4*.*1/qSCl-4*.*1*. One significant SNP (S1_6648896) and three significant SNPs (S1_9324398, S1_9330855 and S1_9332327) on LG1 were mapped to the confidence intervals of QTLs *qSCa-8*.*1/qSCb-8*.*1/qSCl-8*.*1* and *qSCl-8*.*2*, respectively. These comparison results corroborated our findings. Zhang et al. [6] found 4 QTLs (*QTL1-1*, *QTL11-1*, *QTL11-2*, and *QTL13-1*) for sesame seed coat color, however, because of AFLP markers having been mainly used in the study of Zhang et al. in an independent genetic map, it is difficult to determine the relationship of the present loci to them. The remaining SNPs, which were not mapped to the confidence intervals of reported QTLs, indicated the likely existence of new seed coat color-related sites or environment-specific SNPs.

Considering SNPs detected in the most environments with high genetic affect, 4 reliable and stable peaks on 4 LGs were focused on, and 92 candidate genes in the vicinity of 4 significant SNPs were identified. For the 4 SNPs (S1_6648896, S2_12232938, S7_6839839 and S8_8313501), the annotation genes included pentatricopeptide repeat-containing protein (SIN_1006005, SIN_1006010, SIN_1012034), malate dehydrogenase (SIN_1006006), basic helix-loop-helix (BHLH) DNA-binding superfamily protein (SIN_1006020 and SIN_1024895), cytochrome P450 94A2 (SIN_1006022), polyphenol oxidases (SIN_1016759 and SIN_1023237), F-box/LRR-repeat protein 3 (SIN_1023224), etc. SIN_1016759 encodes a predicted polyphenol oxidase (PPO), which participates in the oxidation step in the biosynthesis of proanthocyanidin, lignin, and melanin, and produces black pigments via the browning reaction in plants [42–44]. In sesame, Wei et al. [27] reported that SIN_1016759 was strongly associated with seed coat color, Wang et al. and Wei et al. [15, 18] showed that SIN_1016759 was located in the genomic region of a major QTL for seed coat color. qRT-PCR showed that SIN_1016759 was highly expressed in black sesame seeds from 11 to 20 days but not expressed in white sesame seeds [18], indicating that SIN_1016759 may play an important role in the formation of sesame black coat color. SIN_1023237 encodes a laccase-3 which belongs to multicopper oxidase family [45]. Laccase enzymes were shown to contribute toward cell morphology, secondary cell-wall biosynthesis, and resistance to biotic and abiotic stresses in plant [46]. They also play major roles in proanthocyanidins and lignin deposition and are involved in browning reactions on seed coat pigments [42, 43, 47]. SIN_1006022 encodes a cytochrome P450 protein, and may be related to the formation of seed coat color. Cytochromes P450 play important roles in biosynthesis of flavonoids and their coloured class of compounds, anthocyanins, which are responsible for the pigmentation pattern of vegetative parts and seed [48–51]. SIN_1023226 encodes a WRKY-type transcription factor, which is one of the WRKY family members [52]. The WRKY genes family in flowering plants encode a large group of transcription factors which play essential roles in diverse stress responses, developmental, and physiological processes [53]. SIN_1024895 encodes a bHLH transcription factor. Plant bHLHs are involved in secondary metabolism (including the flavonoid pathway), organ development and responses to abiotic stresses [54–56]. Previous reports have shown that the *WRKY* and *bHLH* genes are involved in regulation of seed coloration [57–60].

## Conclusions

In this study, GWAS for sesame seed coat color were performed using 42,781 SNPs with 366 sesame germplasm lines in 12 environments. GWAS for three color space values, BLUP values from a multi-environment trial analysis and PCs of three color space values identified 224, 119, and 197 significant SNPs, respectively. The 35 significant SNPs detected in more than 6 environments were also detected using the BLUP values. Furthermore, GWAS results for PCs were consistent with those for three color space values. Multiple QTLs reported in previous studies were verified by significant SNPs in the present study, corroborating the GWAS results. Moreover, the most reliable and significant SNPs (S1_6648896, S2_12232938, S7_6839839 and S8_8313501) on 4 different LGs were focused on, and 92 candidate genes were identified. The GWAS showed great power in uncovering genetic variation in sesame seed coat color, and the results will provide new insights into the genetic basis of sesame seed coat color.

## Supporting information

S1 Fig Histograms for the frequency distribution of BLUP values for three color space values. (TIF)

S2 Fig Quantile-quantile plots of observed versus expected–log10(P) values of GWAS results for three seed coat color space values. (TIF)

S3 Fig GWAS for BLUP values of three color space values. (TIF)

S4 Fig GWAS for PCs in twelve environments. (TIF)

S5 Fig Quantile-quantile plots of observed versus expected–log10(P) values of GWAS results for PCs. (TIF)

S1 Table Descriptive statistics of seed coat color across 12 environments. (XLSX)

S2 Table Summary of the first 3 PCs (PC1, PC2, PC3) for three color space values in the dataset of 366 sesame varieties. (XLSX)

S3 Table SNPs significantly associated with three seed coat color space values in more than 6 environments. (XLSX)

S4 Table SNPs significantly associated with PCs scores across more than 6 environments. (XLSX)

S5 Table Candidate genes linked genomic region of SNP most highly associated with seed coat color in sesame. (XLSX)
