# Supplementary figures and images for: Genome-wide association study of seed coat color in sesame (Sesamum indicum L.)
Source: PLoS One. 2021 May 21;16(5):e0251526. doi: 10.1371/journal.pone.0251526 (PMC8139513; doi:10.1371/journal.pone.0251526)

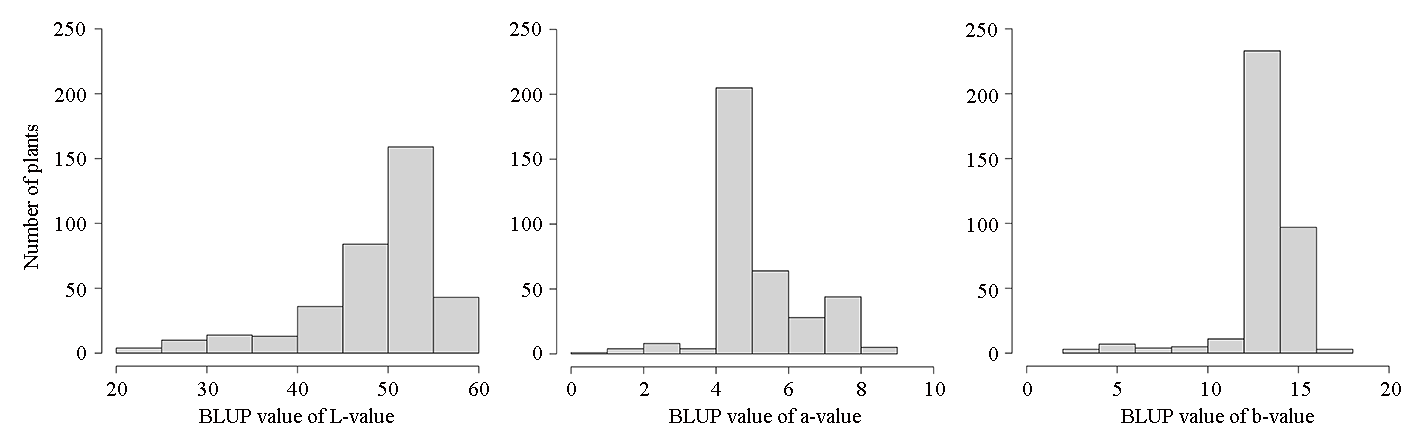

Supplement: S1 Fig — (TIF) [file pone.0251526.s001.tif]

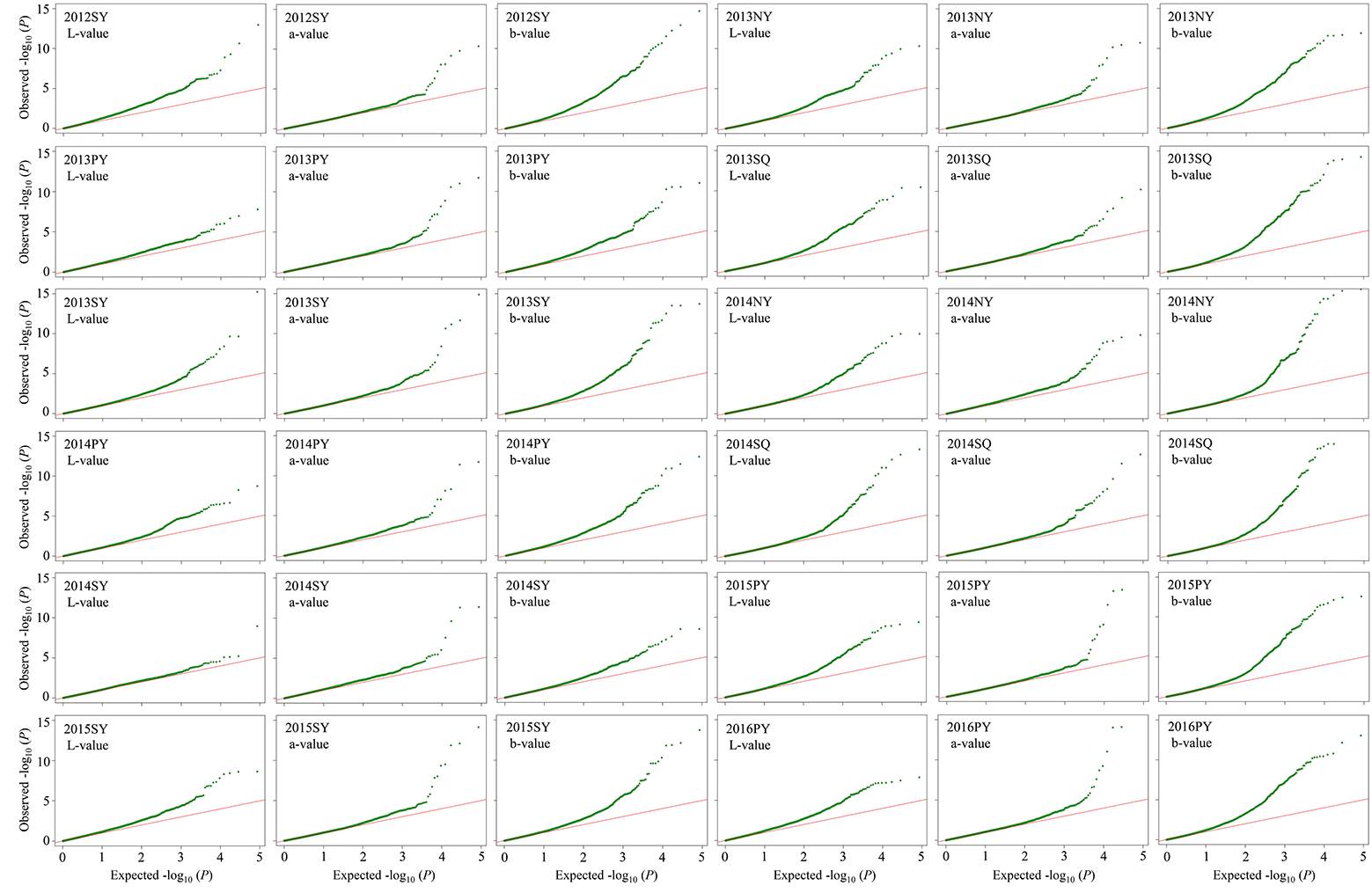

Supplement: S2 Fig — (TIF) [file pone.0251526.s002.tif]

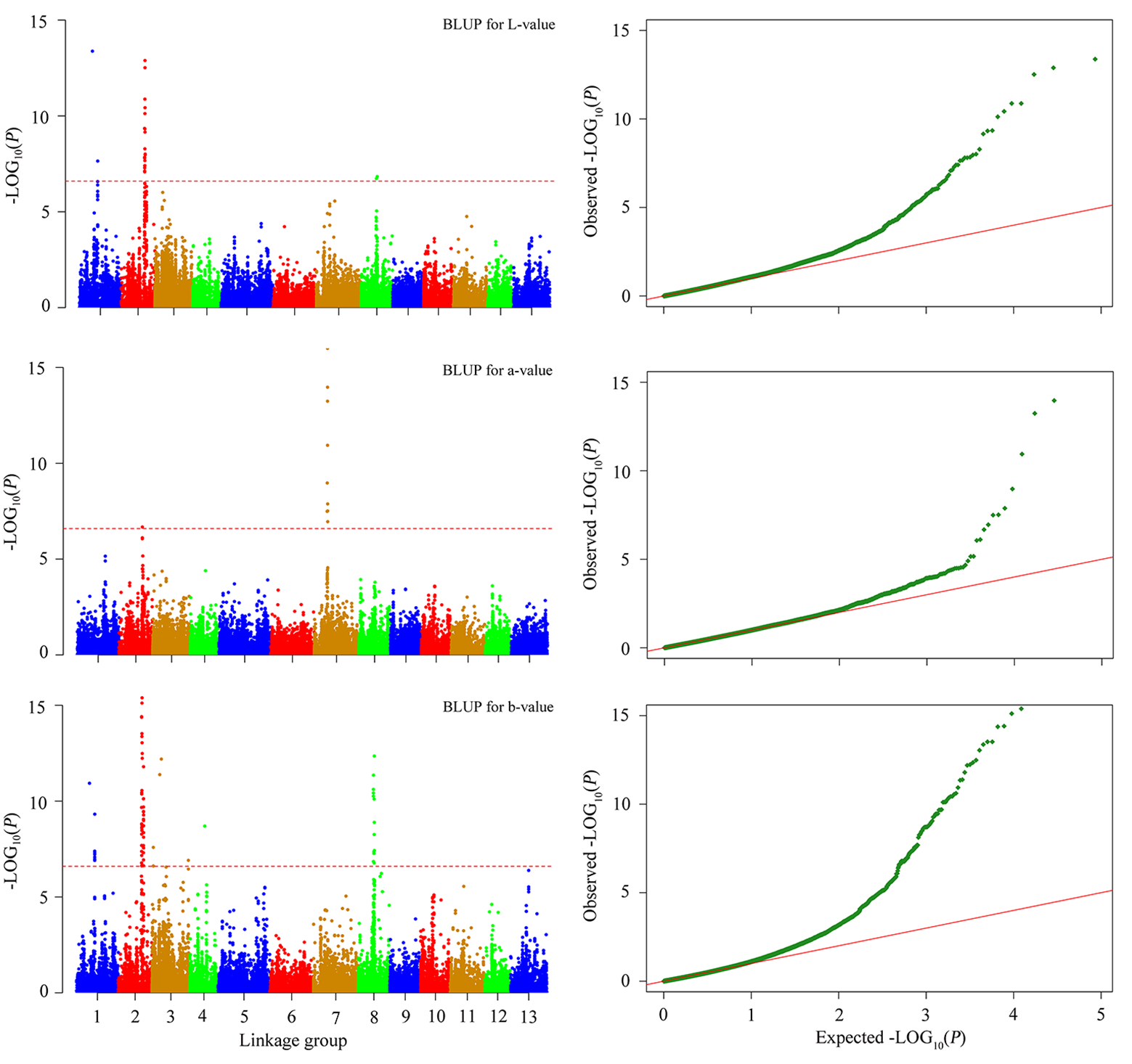

Supplement: S3 Fig — (TIF) [file pone.0251526.s003.tif]

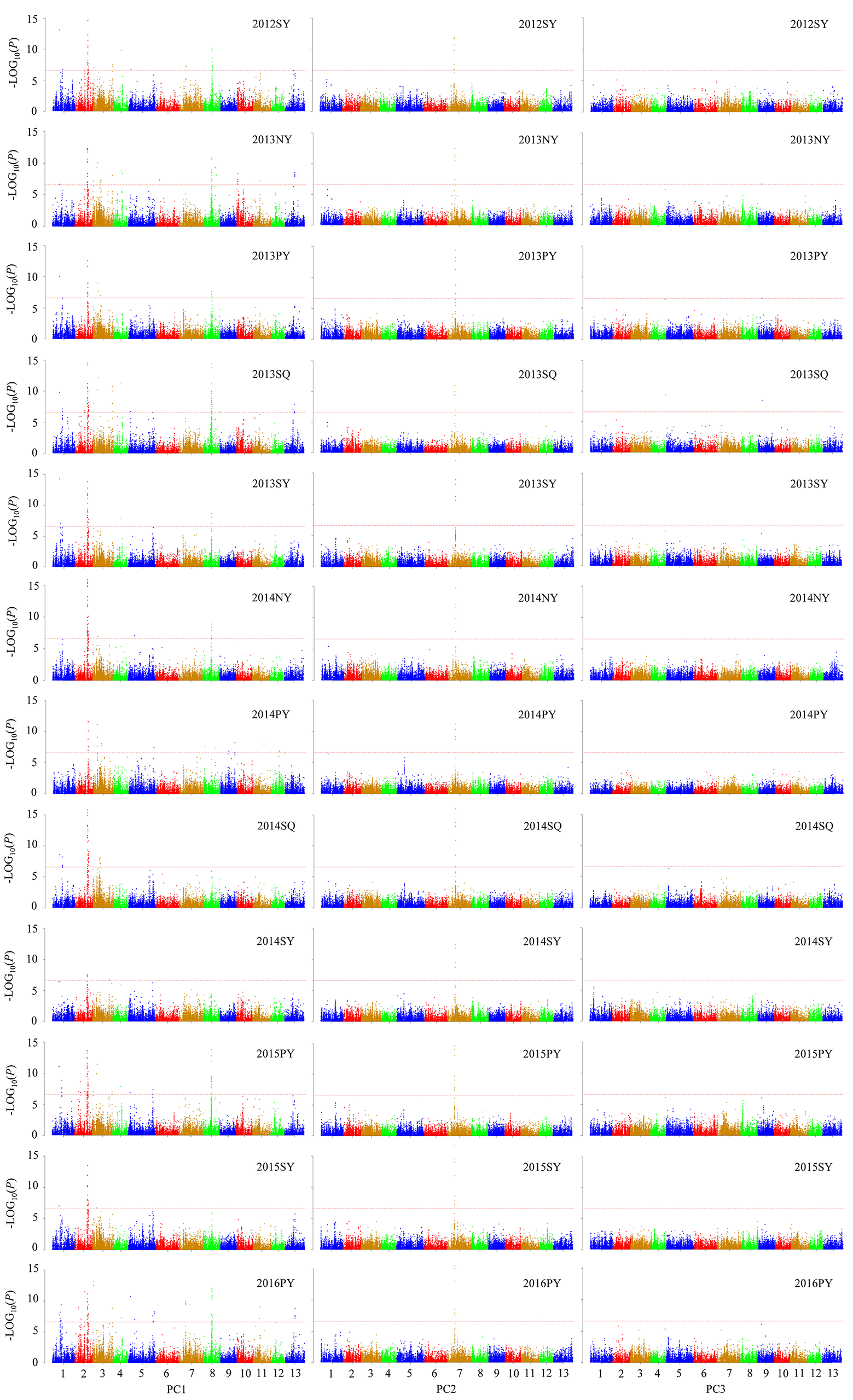

Supplement: S4 Fig — (TIF) [file pone.0251526.s004.tif]

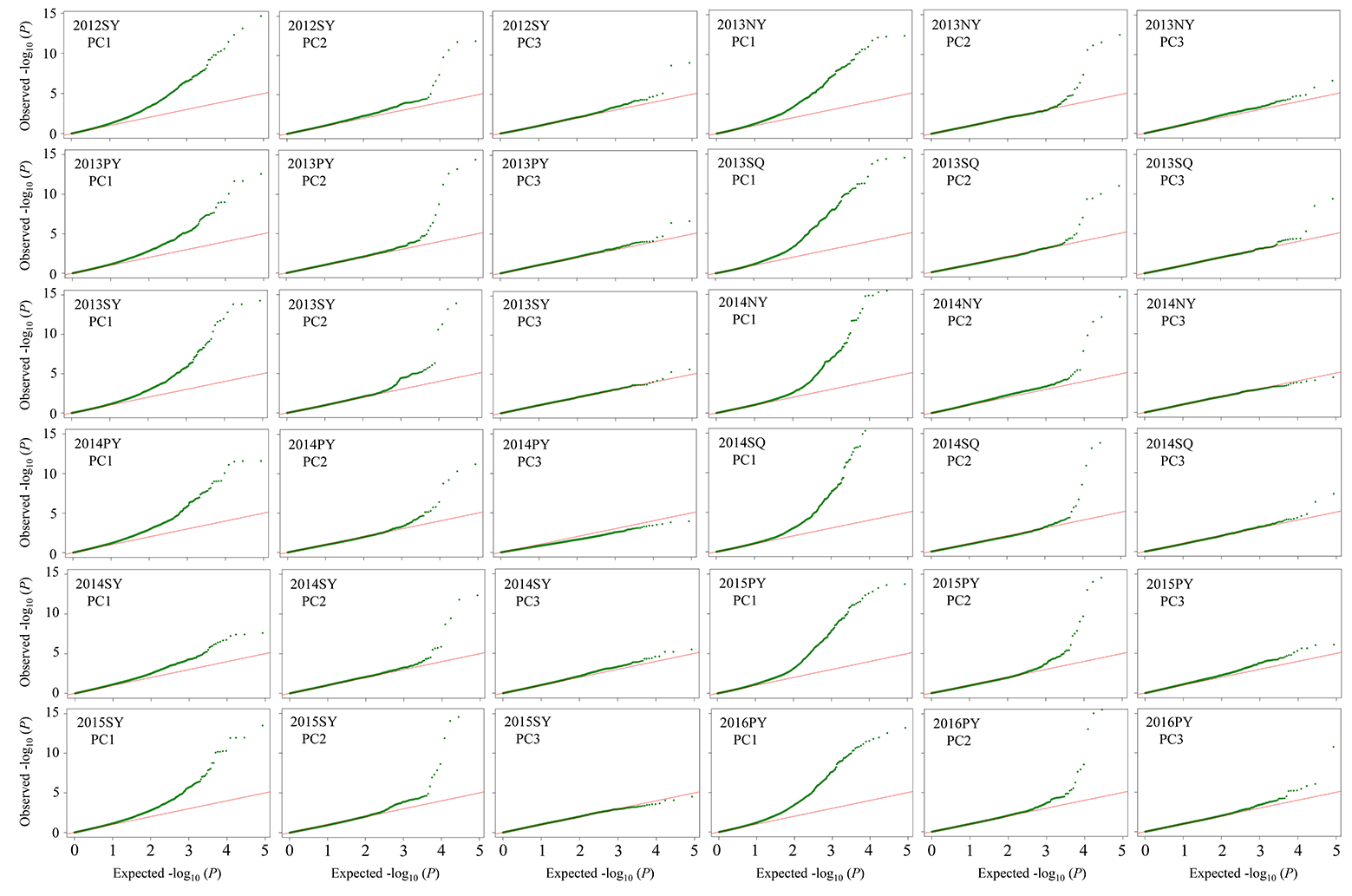

Supplement: S5 Fig — (TIF) [file pone.0251526.s005.tif]
